# Trajectory of self-care behaviour in patients with heart failure: the impact on clinical outcomes and influencing factors

**DOI:** 10.1177/1474515120902317

**Published:** 2020-01-29

**Authors:** Maria Liljeroos, Naoko P Kato, Martje HL van der Wal, Maaike Brons, Marie Louise Luttik, Dirk J van Veldhuisen, Anna Strömberg, Tiny Jaarsma

**Affiliations:** 1 Department of Health, Medicine and Caring Sciences, Linköping University, Linköping, Sweden; 2 Centre for Clinical Research, Sörmland County Council, Uppsala University, Eskilstuna, Sweden; 3 Department of Cardiology, University Medical Centre Groningen, University of Groningen, The Netherlands; 4 Department of Cardiology, University Medical Centre Utrecht, The Netherlands; 5 Research Group Nursing Diagnostics, School of Nursing, University of Applied Sciences Groningen, The Netherlands

**Keywords:** Self-management, depression, heart failure outcomes, self-care behaviour

## Abstract

**Background:**

Patients’ self-care behaviour is still suboptimal in many heart failure (HF) patients and underlying mechanisms on how to improve self-care need to be studied.

**Aims:**

(1) To describe the trajectory of patients’ self-care behaviour over 1 year, (2) to clarify the relationship between the trajectory of self-care and clinical outcomes, and (3) to identify factors related to changes in self-care behaviour.

**Methods:**

In this secondary analysis of the COACH-2 study, 167 HF patients (mean age 73 years) were included. Self-care behaviour was assessed at baseline and after 12 months using the European Heart Failure Self-care Behaviour scale. The threshold score of ⩾70 was used to define good self-care behaviour.

**Results:**

Of all patients, 21% had persistent poor self-care behaviour, and 27% decreased from good to poor. Self-care improved from poor to good in 10%; 41% had a good self-care during both measurements. Patients who improved self-care had significantly higher perceived control than those with persistently good self-care at baseline. Patients who decreased their self-care had more all-cause hospitalisations (35%) and cardiovascular hospitalisations (26%) than patients with persistently good self-care (2.9%, *p* < 0.05). The prevalence of depression increased at 12 months in both patients having persistent poor self-care (0% to 21%) and decreasing self-care (4.4% to 22%, both *p* < 0.05).

**Conclusion:**

Perceived control is a positive factor to improve self-care, and a decrease in self-care is related to worse outcomes. Interventions to reduce psychological distress combined with self-care support could have a beneficial impact on patients decreasing or persistently poor self-care behaviour.

## Introduction

Despite advances in heart failure (HF) treatment and organisation of care, HF outcomes still remains poor, with post discharge mortality rates up to 15%, 20–30% readmission rates within the first 30 days after discharge, and health-related quality of life (HRQoL) poorer than many other chronic conditions.^1,2^

Self-care behaviour is key to enhancing HRQoL and to reduce mortality and morbidity among HF patients, but self-care behaviour remains suboptimal in many patients worldwide.^3–5^ Self-care is a complex process of maintaining health through health-promoting activities and by managing illness, e.g. by exercising, monitoring body weight, taking prescribed medication, and seeking a healthcare provider when symptoms are deteriorating.^6^ Considering the fact that nonadherence with self-care predicts adverse outcomes in HF patients,^2,7,8^ it is vital to identify those patients who are at risk for poor self-care over a longer period. Contributing factors to suboptimal self-care include the difficulty for patients in monitoring signs and symptoms, complex medical regimen, lack of motivation, cognitive decline, and lack of social support.^9^

Numerous educational interventions using different techniques have been tested to improve self-care behaviour in HF patients, such as nurse-led education, using eHealth tools, goal setting, the use of symptom diaries, and home-based telemonitoring.^3,10,11^ Most of these studies report that patients’ self-care behaviour improved after the intervention, but decreased in the long term unless they received continual self-care support.^12^ So far, only a few studies have examined trajectories of self-care behaviour among HF patients, and in these studies the longest follow-up period was 6 months.^2,13^

Adequate self-care behaviour is shown to predict a reduced risk of hospitalisations and mortality,^8,14^ but no studies have reported the relationship between the trajectory of self-care behaviour and subsequent clinical outcomes among HF patients. A recent review described depression as consistently associated with poor self-care behaviour, whereas there was a discrepancy in the association of self-care with age, sex, education, and left ventricular ejection fraction (LVEF).^15^

However, these factors are mainly identified from studies using a cross-sectional design, and trajectories of self-care over time were not the main focus.^15^ It is therefore unknown which factors are related to decreased or increased self-care behaviour, and which factors contribute to HF patients continuing their necessary self-care over time.

Before trying to design more effective interventions to improve patients’ ability to perform effective self-care, it is important to know which factors determine long-term management of self-care. The purpose of this study was therefore (1) to describe the trajectory of HF patients according to changes in self-care behaviour, (2) to examine the relationship between changes of self-care and subsequent clinical outcomes over time, and (3) to identify factors related to change in self-care behaviour.

## Method

### Design and settings

This study is a secondary data analysis using data from a randomised controlled intervention study, the Coordinating Study Evaluating Outcomes of Advising and Counselling in Heart Failure (COACH)-2 study.^16^ For this secondary analysis a cross-sectional and longitudinal design was used. After optimisation of the medical management and patient education in HF and related subjects, patients were randomly assigned to follow-up by a general practitioner (GP) in primary care or at an outpatient HF clinic for 12 months. The long-term results showed no differences between the two groups regarding guideline adherence for medication, and patients’ medication adherence or level of healthcare use.^17^ The investigation conforms with the principles outlined in the Declaration of Helsinki and is listed in the Netherlands Trial Register (NTR1729).

### Study participants

Eligible participants were patients with HF who were (1) clinically stable, (2) optimally up-titrated on medication according to ESC guidelines,^18^ and (3) had received optimal education and counselling on pre-specified issues regarding HF and its treatment. Patients who met the inclusion criteria were recruited from four outpatient HF clinics in the Netherlands between November 2009 and April 2012, as reported previously.^16,17^ Written informed consent was obtained from all participants.

In the current study, we excluded patients from the analysis in cases where they were lost or died during follow-up. Their self-reported self-care behaviour at the 12-month follow-up was lacking, and the patients could not be classified according to their changes of self-care behaviour. Sample size calculation for the main study has been reported previously, with 100 patients in each arm (follow-up by a primary care or at an outpatient HF clinic) were considered to be necessary.^16^

### Measurements and data collection

Data were collected with validated self-administered questionnaires and from the patients’ medical record at baseline and at 12 months’ follow-up. Both at baseline and follow-up the self-administered questionnaires were handed to the patients during a HF clinic visit and completed during that visit, without any interference of the HF nurse or study personnel.

HF-specific self-care, HRQoL, perceived control, and depressive symptoms were assessed by the following measurements at baseline and at 12 months. To assess HF-specific self-care behaviour, the nine-item European Heart Failure Self-care Behaviour (EHFScB) scale was used.^19^ It is a valid and reliable scale used worldwide. Each item was rated by five response options ranging from 1 (I completely agree) to 5 (I don’t agree at all). The total score was calculated and standardised from 0 to 100, with higher scores reflecting better self-care. A threshold score of ⩾70 was used to define self-care behaviour as good and <70 as poor self-care.^20^ Patients were classified into four self-care behaviour groups according to the threshold score at baseline and at the end of follow-up: poor–poor, poor–good, good–poor, and good–good. When patients did not respond to the questionnaire, self-care of the patients was classified into poor self-care behaviour (EHFScB scale score <70).

HRQoL was assessed using the EuroQoL visual analogue scale (EQ VAS).^21^ Patients were asked to rate their health status on a 20-cm vertical scale with end points of 0 (the worst health) and 100 (the best health). Level of knowledge regarding HF and HF symptoms was evaluated using the Dutch HF knowledge scale.^22^ This is a self-administered 15-item valid and reliable scale, with a higher score indicating higher level of HF knowledge (range 0–15).

Perceived control was evaluated by the Control Attitudes Scale. This scale measures the degree to which patients feel they have control and conversely helplessness related to their cardiac disease. The total scores range from 4 to 28. A higher score on the scale indicates higher feelings of control. Reliability and validity have been assessed in patients with HF.^23^

Depressive symptoms were assessed using the Center for Epidemiologic Studies Depression Scale (CES-D).^24^ The CES-D is a 20-item self-report questionnaire and total scores range from 0 to 60, with a higher scores indicating more severe depressive symptoms. A cut-off point of 16 is commonly used to identify those with depression.

The following demographic and clinical variables of patients were collected from the questionnaires and medical records: age, sex, marital status, education, aetiology of HF, duration of HF, admission in past 6 months prior to inclusion, New York Heart Association (NYHA) functional class, heart rate, LVEF, NT-pro B-type natriuretic peptide, estimated glomerular filtration rate (GFR), comorbidity, and medication.

### Clinical outcomes

Data were collected from the medical chart on rehospitalisation due to cardiovascular (CV) reasons: mortality, rehospitalisation, and emergency visits for any reasons. In the current study, hospitalisation included unplanned and planned hospital admissions. A planned hospitalisation was defined as a hospitalisation to receive planned intensive treatment, such as cardiac resynchronisation therapy (CRT)/implantable cardioverter defibrillator (ICD) implantations, and percutaneous coronary intervention (PCI), because these therapies might influence patients’ subsequent mortality and morbidity. Researchers from the original COACH-2 study discussed and adjudicated whether it was an unplanned or planned hospitalisation, including reasons for hospitalisations and emergency visits based on the medical records.

### Statistical analysis

In all analysis in this secondary analysis, patients were analysed as one group since there were no differences between the group who were followed-up at a HF clinic or in primary care. The second author (NK) performed all analysis. Categorical data are presented as frequencies and percentages. For continuous variables with a normal distribution, the mean and SDs are reported. For variables not normally distributed, the median and interquartile ranges (IQRs) are reported. Student’s t-test or the one-way analysis of variance (ANOVA) was used for comparison of normally distributed continuous data, and Mann–Whitney U-test or the Kruskal–Wallis test was used for non-normally distributed continuous data. Categorical variables were compared with the χ^2^ test or Fisher’s exact test as appropriate. When there was likely to be difference in the continuous variables among groups (*p* < 0.07), we performed post hoc comparisons using Dunnett’s method for continuous variables with a normal distribution, or Bonferroni correction for continuous variables with a non-normal distribution and categorical variables (*p* < 0.017). Kaplan–Meier curves and log-rank tests were performed to compare survival curves of the four self-care groups. Cox regression analysis was conducted to assess relationships between the self-care groups and subsequent clinical outcomes.

Missing data from the EHFScB scale were handled according to the recommendations of the constructors of the scale: if fewer than three items of the total score were missing, these items were substituted with a score of 3. If more than three items were missing, the EHFScB scale was considered missing. For missing values in other instruments, missing items were substituted with a mean score calculated using the rest of the items in cases where up to 50% of items were missing. Patients who died before the follow-up were excluded from the analysis.

All statistical tests were two-tailed, and statistical significance was defined as *p* < 0.05. All analyses were performed with SAS version 9.4 for Windows (SAS Institute Inc., Cary, NC, USA).

## Results

### Participant characteristics

**Figure 1.**
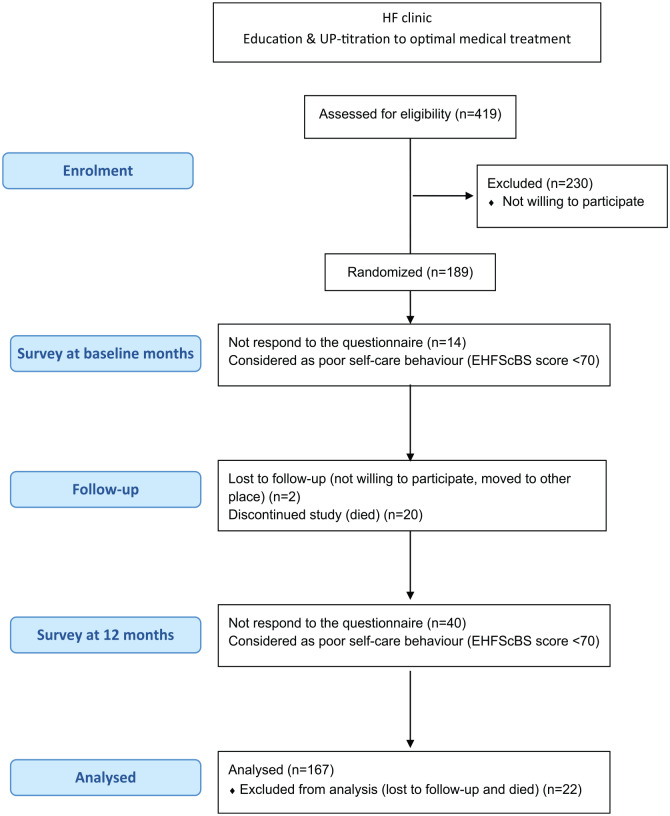
Flow diagram of the study.

As previously reported,^17^ 419 patients met the inclusion criteria and 189 patients were randomised and followed-up for 12 months (see Figure 1). During the 12 months, two patients were lost to follow-up, because one patient no longer wanted to participate in the study and the other patient moved to another place. Twenty patients (11%) died, of which seven died due to CV reasons (*n* = 7). Thus, 22 patients (12%) were excluded from all analysis in the current study.

The mean age of the patients included in the present study (*n* = 167) was 72 years, 38% were female, and approximately 60% of patients were married or had a partner (Table 1). The median duration of HF diagnosis was just less than 2 years, and mean LVEF was 31% at the time of diagnosis. The mean score of the EHFScB scale was 80.1±18.2 at baseline (*n* = 153) and 76.8±18.0 at the end of follow-up (*n* =127).

**Table 1.** Characteristics of study patients at baseline.

	All (*N* = 189)	Study patients included (*N* = 167)
***Demographics***		
Age, years	72.5±11.0	71.9±11.2
Sex, female	72 (38%)	64 (38%)
Marital status		
Single	17 (9.3%)	17 (10%)
Married or have a partner	104 (57%)	92 (57%)
Divorced or widowed	60 (33%)	51 (31%)
Education		
Elementary school, 6 years	44 (24%)	41 (25%)
Education after elementary school	115 (63%)	99 (61%)
University or higher professional education	21 (12%)	21 (13%)
Follow-up by primary care only (not in HF clinic)	97 (51%)	85 (51%)
***Clinical characteristics***		
Ischemic aetiology	90 (48%)	75 (45%)
Duration of HF, days	693 (388–1541)	716 (388–1514)
Admission in past 6 months	17 (9%)	14 (8.4%)
NYHA class, I or II	163 (86%)	149 (89%)
Heart rate (bpm)	70.2±14.0	69.9±14.2
LVEF (%) at diagnosis	31.2±8.7	31.4±8.7
NT-pro BNP (ng/L) median (Q1-Q3)	1031 (406–1870)	967 (313–1766)
GFR (mL/min/1.73m^2^)	57.2±18.5	58.2±18.8
Myocardial infarction	78 (41%)	64 (38%)
History of atrial fibrillation	78 (41%)	67 (40%)
Diabetes (type I and II)	43 (23%)	36 (22%)
COPD	35 (19%)	28 (17%)
***Medication and device therapy***		
ACEI/ARB	173 (92%)	155 (93%)
Beta-blocker	174 (92%)	157 (94%)
Mineralocorticoid receptor antagonist	91(48%)	81 (49%)
Implantable cardioverter defibrillator	24 (13%)	24 (14%)
CRT/CRT-D	8 (4.2%)	7 (4.2%)
Pacemaker	5 (2.7%)	5 (3.0%)
***Psychological characteristics***		
Perceived control score (range 4–28)	18.6±5.1	18.9±4.9
CES-D score (range 0–60)	6.4±4.6	6.4±4.6
Depression (CES-D score ⩾16)	6 (3.6%)	6 (4.0%)
Quality of life (range 0–100)	72.8±14.1	73.4±14.2
Dutch HF Knowledge score (range 0–15)	12.4±2.0	12.3±2.0
EHFScBS score (range 0–100)	80.1±17.9	80.1±18.2

Values show mean±SD or *n* (%).

HF, heart failure; NYHA, New York Heart Association; LVEF, left ventricular ejection fraction; NT-pro BNP, N-terminal pro b-type natriuretic peptide; GFR, glomerular filtration rate; COPD, chronic obstructive pulmonary disease; ACEI, angiotensin-converting-enzyme inhibitor; ARB, angiotensin II receptor blocker; CRT-(d) cardiac resynchronisation therapy (defibrillator); CES-D, Center for Epidemiologic Studies Depression scale.

Compared with the patients included in the present study, excluded patients were likely to have more NYHA III or IV (11% vs. 36%, *p* < 0.001), higher BNP levels (median, 967 ng/dL vs. 1302 ng/dL, *p* = 0.030), history of myocardial infarction (38% vs. 64%, p = 0.023), and lower perceived control score (18.9±4.9 vs. 16.3±6.0, *p* = 0.027).

### Trajectory of self-care behaviour

**Figure 2.**
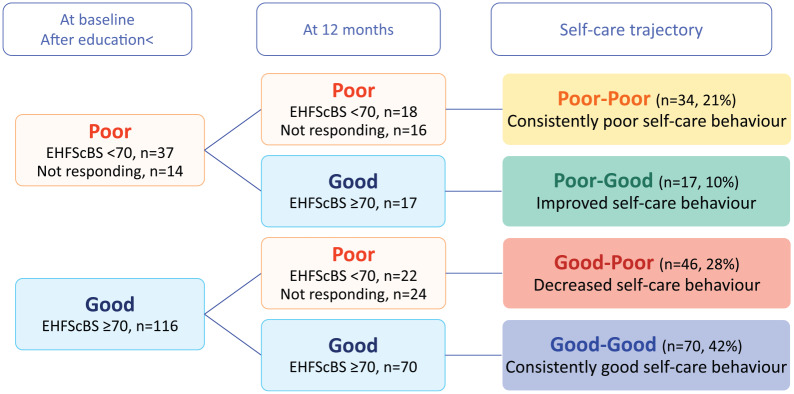
Trajectory of self-care behaviour.

The 167 patients were classified into four groups as follows (Figure 2 and supplementary table). At baseline, 70 patients persistently had good self-care behaviour assessed by the EHFScBS ⩾ 70 (good–good group, 42%), 37 patients had the EHFScBS score of less than 70, and 14 patients did not reply to the first questionnaire. Therefore, the 51 patients (31%) were classified as having poor self-care behaviour at baseline. Among the 51 patients, 18 patients had EHFScBS score <70 at 12 months and 16 patients did not reply the second questionnaire. These 34 patients were classified into a consistently poor self-care behaviour group (poor–poor group, 21%). On the other hand, 17 patients improved their self-care behaviour at 12 months (poor–good group, 10%). Meanwhile, 116 patients had good self-care behaviour (EHFScBS ⩾70) at baseline, and 22 patients decreased their level of self-care (the EHFScBS score <70) at 12 months and 24 patients did not respond to the second questionnaire. These 46 patients were classified into a decreased self-care behaviour group (good–poor group, 28%).

### Trajectory of self-care behaviour and clinical outcomes

During the 12 months follow-up period, 34 patients of the 167 (20%) had hospitalisations for any reasons. Twenty patients were hospitalised due to CV reasons (12%), and 6 patients (3.6%) were for HF. Among the 20 patients, three patients had planned CV hospital admissions, in which 2 patients had a CRT-D implantation and one patient received PCI. One patient who had hospitalisation because of acute HF afterwards received an ICD. Eighteen patients (11%) were hospitalised for non-CV reasons during a 1-year follow-up.

**Figure 3.**
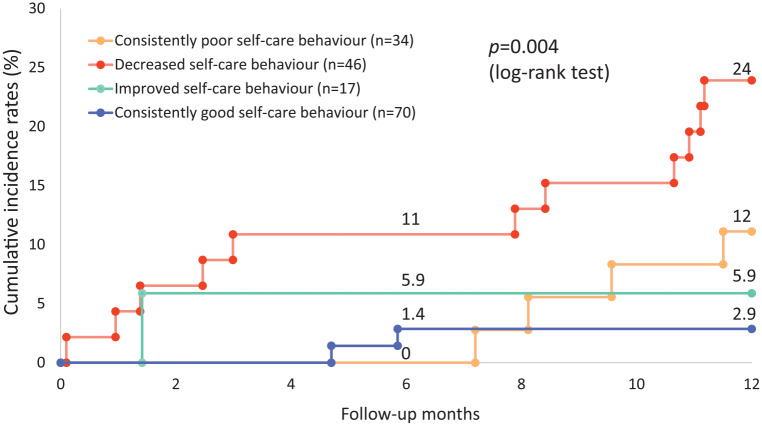
Cumulative incidence rates of hospitalisation for cardiovascular reasons. *p*=0.004 (*p*=0.012 after Bonferroni correction) by log rank test, decreased self-care behavior group vs. consistently good self-care group, Hazard ratio 9.29, 95% confidence interval [2.06-41.93], *p*=0.004 *p*=0.061 by log-rank test, consistently poor self-care behavior group vs. consistently good self-care behavior group *p*=0.072 by log-rank test, improved self-care behavior group vs. consistently good self-care behavior group.

The cumulative incidence rates of CV hospitalisations were significantly different among the four groups (*p* = 0.004, Figure 3). Cox regression analysis showed that patients with decreasing self-care behaviour (good–poor group) had a nine-times higher risk of hospitalisation for CV reasons compared to patients with persistently good self-care behaviour (good–good group, hazard ratio (HR) 9.29, 95% confidence interval (CI) =2.06–41.93, *p* = 0.004). This result also remained after adjustment for the random allocation of primary care follow-up, marital status, NYHA functional class, and the perceived control score at baseline (HR = 11.29, 95% CI = 2.44–52.29, *p* = 0.002).

**Figure 4.**
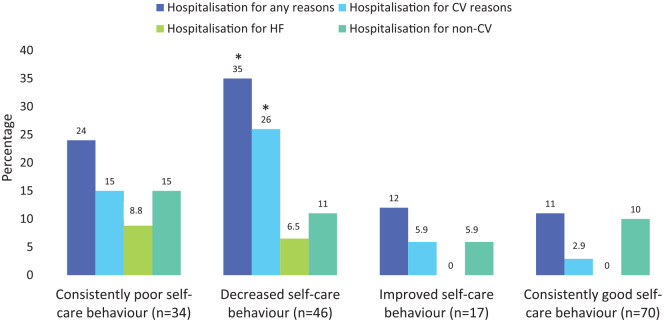
Trajectory of self-care behaviour and hospitalisations. *p<0.05 after Bonferroni correction in a comparison with a consistently good self-care group.

Figure 4 shows the relationship between self-care trajectories and any hospitalisations.

Patients with decreasing self-care behaviour (good–poor group) had significantly higher all-cause hospitalisation rates (35% vs. 11%, *p* = 0.002) and for CV reasons (26% vs. 2.9%, *p* < 0.001) compared to the reference group (good–good group). Patients with consistently poor self-care behaviour (poor–poor group) did not have significantly higher rates of CV hospitalisation (15% vs. 2.9%, *p* = 0.036, after the Bonferroni *p* = 0.108) and HF hospitalisations (8.8% vs. 0%, *p* = 0.033, after the Bonferroni *p* = 0.099) compared to the reference group (consistently good self-care), after Bonferroni correction. The number of all-cause hospitalisations and CV hospitalisations in the good–poor group were also significantly higher than the reference group (all *p* < 0.05 after the adjustment, Table 2). There were no significant differences in emergency room visits.

**Table 2.** Quality of life and clinical outcomes at 12 months follow-up (*N* = 167).

	Poor–poor (*n* = 34)	Good–poor (*n* = 46)	Poor–good (*n* = 17)	Good–good (*n* = 70)	*p*-value
Quality of life, *n* = 125	64.9±16.3	70.0±12.9	78.5±13.8	72.0±11.9	0.022
***Total number of hospitalisations, n (%)***					
Hospitalisations for any reason, once	6 (17%)	11 (24%)*	1 (5.9%)	7 (10%)	0.054
⩾2	2 (5.9%)	5 (11%)	1 (5.9%)	1 (1.4%)	
Hospitalisations for CV reason, once	4 (12%)	11 (24%)*	1 (5.9%)	2 (2.9%)	0.003
⩾2	1 (2.9%)	1 (2.1%)	0 (0%)	0 (0%)	
HF hospitalisations for HF, once	2 (5.9%)	3 (6.5%)	0 (0%)	0 (0%)	0.058
⩾2	1 (2.9%)	0 (0%)	0 (0%)	0 (0%)	
Hospitalisations for non-CV, once	4 (12%)	2 (4.4%)	0 (%)	6 (8.6%)	0.453
⩾2	1 (2.9%)	3 (6.5%)	1 (5.9%)	1 (1.4%)	
***Emergency room visits, n (%)***					
Visits for any reasons	6 (18%)	8 (17%)	3 (18%)	6 (8.6%)	0.377
Visits for CV reasons	4 (12%)	3 (6.5%)	1 (5.9%)	2 (2.9%)	0.274
Visits for HF	1 (2.9%)	0 (0%)	0 (0%)	0 (0%)	0.305
Visits for non-CV	3 (8.8%)	5 (11%)	2 (12%)	4 (5.7%)	0.700
***Total number of emergency room visits, n (%)***					
Visits for any reasons, once	4 (12%)	4 (8.7%)	2 (12%)	6 (8.6%)	0.200
⩾2	2 (5.9%)	4 (8.7%)	1 (5.9%)	0 (0%)	
Visits for CV reasons, once	4 (12%)	3 (6.5%)	1 (5.9%)	2 (2.9%)	0.274
Visits for HF, once	1 (2.9%)	0 (0%)	0 (0%)	0 (0%)	0.305
Visits for non-CV, once	1 (2.9%)	1 (2.2%)	1 (5.9%)	4 (5.7%)	0.151
⩾2	2 (5.9%)	4 (8.7%)	1 (5.9%)	0 (0%)	

Note: Hospitalisation included unplanned hospitalisation and a planned hospitalisation for an intensive treatment such as CRT/ICD implantation and PCI that might influence patients’ mortality and morbidity.

* Post-hoc test, *p* < 0.017 by comparison of hospitalisations with a group of good–good self-care. When a *p*-value was less than 0.017, the *p*-value achieved the statistically significant after Bonferroni correction. The statistical threshold was adjusted to 0.05/3 = 0.017.

CV, cardiovascular; HF, heart failure; CRT, cardiac resynchronisation therapy; ICD, implantable cardioverter defibrillator.

Although the QoL score in the good–good self-care group was comparable to the scores in the other three groups, the QoL score of patients with improved self-care behaviour (78.5±13.8) was higher than patients with consistently low self-care behaviour (64.9±16.3, *p* < 0.05 after the Bonferroni adjustment) at 12 months. There were no significant changes in the QoL scores between baseline and 12 months in all self-care groups.

### Patients’ characteristics according to the trajectory of self-care behaviour

In total, 40% of patients with consistent low self-care and 50% of patients with decreasing self-care were followed by primary care. Whether patients were followed by primary care or at the HF clinic did not influence changes in self-care behaviour significantly (Table 3).

**Table 3.** Characteristics of patients classified by changes of self-care behaviour (*N* = 167).

	Poor–poor (*n* = 34)	Good–poor (*n* = 46)	Poor–good (*n* = 17)	Good–good (*n* = 70)	*P*-value
Follow-up by primary care only, *n* (%)	14 (41%)	23 (50%)	8 (47%)	40 (57%)	0.477
***Demographics***					
Age, years	72±12	72±14	69±7.7	72±10	0.691
Sex, female, *n* (%)	15 (44%)	19 (41%)	5 (29%)	25 (36%)	0.699
Marital status, *n* (%)					**0.028**
Single	3 (8.9%)	4 (9.1%)	1 (5.9%)	9 (13%)	
Married or have a partner	15 (47%)	20 (45%)	14 (82%)	43 (62%)	
Divorced or widowed	14 (41%)	20 (45%)	2 (12%)	17 (25%)	
***Clinical characteristics***					
Ischemic aetiology, *n* (%)	13 (38%)	21 (46%)	10 (59%)	31 (44%)	0.580
Duration of HF, days, median (Q1–Q3)	1070 (430–2025)	669 (401–1454)	1357 (614–1952)	572 (313–1190)	**0.044**
NYHA class, I or II, *n* (%)	27 (79%)	39 (85%)	17 (100%)	66 (94%)	**0.038**
LVEF (%) at diagnosis	31.6±9.4	32.6±9.0	30.9±8.5	30.6±8.3	0.676
GFR (mL/min/1.73m^2^)	54.6±20.3	63.2±19.8	61.3±23.5	55.8±14.3	0.234
Diabetes, *n* (%)	6 (18%)	13 (28%)	5 (29%)	12 (17%)	0.392
***Medication and device therapy at baseline***					
ACEI/ARB	33 (97%)	40 (87%)	16 (94%)	66 (94%)	0.366
Beta-blocker	31 (91%)	44 (96%)	17 (100%)	65 (93%)	0.678
Mineralocorticoid receptor antagonist	19 (56%)	22 (48%)	10 (59%)	30 (43%)	0.500
ICD	3 (8.8%)	6 (13%)	5 (29%)	10 (14%)	0.292
CRT/CRT-D	5 (15%)*	0 (0%)	1 (5.9%)	1 (1.4%)	**0.006**
Pacemaker	2 (5.9%)	2 (4.4%)	0 (0%)	1 (1.4%)	0.487
***Psychological characteristics***					
At baseline					
Perceived control score, *n* = 162	17.0±3.8	19.3±4.9	22.0±3.5*	18.8±5.3	**0.009**
Depression (the score ⩾16), n=152	0 (0%)	2 (4.4%)	0 (0%)	4 (5.9%)	0.856
Dutch HF Knowledge score, *n* = 141	11.4±2.6	12.6±1.8	11.9±2.4	12.5±1.8	0.080
EHFScBS score, *n* = 153	55.0±10.0*	86.6±9.1	50.4±19.2*	90.0±8.0	**<0.001**
Quality of life, *n* = 146	70.3±13.6	75.2±12.8	75.4±15.6	70.3±13.6	0.515
At 12 months					
Perceived control score, *n* = 145	16.7±4.4*	18.9±4.1	19.6±4.8	19.4±4.5	0.069
Depression (the score ⩾16), *n* = 128	4 (21%) †	5 (23%) †	1 (5.9%)	6 (8.6%)	0.164
Dutch HF Knowledge score, *n* = 119	11.5±2.7	12.5±1.9	12.4±1.1	12.4±1.5	0.254
EHFScBS score, *n* = 127	55.6±10.0*	54.0±16.4*	84.3±5.6	87.7±8.0	**<0.001**

* Post-hoc test, *p* < 0.017 by comparison of hospitalisations with a group of good–good self-care. When a *p*-value was less than 0.017, the *p*-value achieved the statistically significant after Bonferroni correction. The statistical threshold was adjusted to 0.05/3 = 0.017.

† *p* < 0.05, at baseline vs. at 12 months.

HF, heart failure; NYHA, New York Heart Association; LVEF, left ventricular ejection fraction; IQR, interquartile range; NT-pro BNP, N-terminal pro b-type natriuretic peptide; GFR, glomerular filtration rate; COPD, chronic obstructive pulmonary disease; ACEI, angiotensin-converting-enzyme inhibitor; ARB, angiotensin II receptor blocker; MRA, mineralocorticoid receptor antagonist; CES-D, Center for Epidemiologic Studies Depression scale.

**Consistent good self-care behaviour:** Mean age of patients in the good–good group was 72 years old and 36% were female. Median duration of HF was approximately 18 months and most patients had mild HF (94%, NYHA class I or II). Only one patient received CRT therapy.

**Improved self-care behaviour**: Compared with the reference group (good–good group), patients who improved self-care behaviour (poor–good group) had a significantly higher score of perceived control at baseline (18.8±5.3 vs. 22.0±3.5, *p* < 0.05 after the adjustment), and they were likely to have longer duration of HF, although it did not reach a statistically significant level after adjustment (median 572 days vs. 1357 days, *p* = 0.036, after the Bonferroni correction, *p* = 0.108).

**Consistent poor self-care behaviour:** Compared with the reference group, patients in the poor–poor group received significantly more CRT therapy (1.4% vs. 15%, *p* = 0.014) and were less likely to have NYHA class I or II (95% vs 81%, *p* = 0.037, after the Bonferroni correction, *p* = 0.126) and likely to have longer duration of HF (median 572 days vs. 1070 days, *p* = 0.028, after the Bonferroni correction, *p* = 0.084), although these did not reach a statistically significant level after adjustment. After 12 months their perceived control score (16.7 ± 4.4) was significantly lower than the reference group (19.4± 4.5, *p* < 0.05 after adjustment); however, no differences were found in HF knowledge scores between the groups. At baseline none of patients had depressive symptoms on, but 21% suffered from depression at 12 months in the poor–poor group (*p* = 0.029).

**Decreased self-care behaviour**: Patients who decreased their self-care (good–poor) were more likely to live alone (single, divorced or widowed) than the reference good–good group, but it did not reach a statistically significant level (55% vs 38%, *p* = 0.078). HF knowledge scores of patients in the good–poor group were comparable to the reference group. Compared with baseline, more patients in this group had depressive symptoms at 12 months (4.4% vs. 22%, *p* = 0.032).

When excluding 48 patients (28%) who did not respond to their questionnaires from our analysis, 121 patients were classified according to the their self-care levels [16 patients (13%) in a poor–poor group, 22 patients (18%) in a good–poor group, 13 patients (11%) in a poor–good group, and 70 patients (58%) were a good–good group.] Patients with decreased self-care behaviour had a significantly higher hospitalisation rate due to CV reasons (23% vs. 2.9%, *p* = 0.003) than patients with consistently good self-care behaviour. The decreased self-care behaviour remained an independent predictor for CV hospitalisation after adjustment for the random allocation of primary care follow-up and NYHA functional class, HR =14.25, 95% CI = 2.75–73.83, *p* = 0.002). On the other hand, there were no significant differences in perceived control scores between a poor–good group and a good–good group, and the prevalence of depression at baseline and at 12 months was comparable among all self-care groups after excluding the non-responded patient.

## Discussion

To our knowledge, this is the first study focused on the trajectory of self-care behaviour and clinical outcomes in a large sample of HF patients. Our major findings were that (1) one in five patients had persistently poor self-care behaviour despite self-care education and follow-up at an HF clinic or by a GP, and a third of the patients decreased in self-care behaviour, (2) patients who decreased in self-care had worse clinical outcomes and (3) the presence of depression and low perceived control were factors significantly related to poor or decreased self-care behaviour.

It is noteworthy that 27% of patients decreased self-care behaviour over time and their hospitalisation rates were significantly higher than patients persistently having good self-care behaviour, regardless of the fact that there were no significant differences in age, HF severity and levels of HF knowledge between the two groups. Patients who decreased their self-care had lower perceived control, and increased depression at 12 months. These results point out the challenges for HF patients to continue to perform adequate self-care over time and also underline the impact of depressive symptoms and perceived control in promoting self-care.^25^

Although all patients received HF education and were followed-up by primary care or a HF clinic, 21% had persistently poor self-care behaviour and approximately 20% were hospitalised due to any reasons. Similar to patients with decreasing self-care, these patients had also lower perceived control, and one in five patients had depressive symptoms at 12 months. In a study reported by Hwang et al.,^26^ HF patients who had high knowledge, but performed poor self-care tended to have more depressive symptoms and lower perceived control than patients with high knowledge who performed good self-care.

Given that both patients with persistent poor self-care and patients with decreasing self-care had HF knowledge comparable to patients who persistently had good self-care, a basic educational intervention aimed to enhance knowledge would not be helpful for these patients. More importantly, both these groups of patients increased depressive symptoms and had low perceived control. Depression is known to be a risk factor for poor self-care,^27^ and it may interfere with patients’ ability to learn and make decisions on how to deal with symptoms, and also it may reduce patients’ motivation to engage in self-care. In prior studies HF knowledge was not related to perceived control,^28^ and interventions focused on attitude and barriers were effective in improving perceived control.^29^ Heo et al. reported recently that a comprehensive meditation intervention,^30^ combining mindfulness and compassionate mediation and self-management, reduced depressive symptoms and increased perceived control, social support and HRQoL. Accordingly, for HF patients who persistently report poor self-care behaviour as well as patients who decrease self-care, a holistic intervention aimed at decreasing psychosocial distress and improving self-care might have a beneficial impact.

Ten per cent of patients increased their self-care behaviour, and they had higher perceived control at baseline, and a higher QoL score at 12 months. Lower levels of perceived control were shown to affect physical and depressive symptoms and HRQoL negatively.^28^ Our findings confirm the previous results and suggest that higher perceived control could be a positive factor for promoting good self-care behaviour and maintaining better QoL. As previously been found, low perceived control is associated with poorer self-care activities and is independently associated with physical and mental health status. If a person can increase/maintain control, then they are more likely to manage self-care, which can improve both physical and mental health status.^28^

Forty per cent of patients persistently had good self-care behaviour. These patients had stable HF and could maintain good perceived control. None of the patients had hospitalisations for HF. Thus, a standard HF management approach by HF nurses or primary care would fit this population.

Finally, it is also worth noting that trajectories of self-care behaviour were not influenced by the follow-up methods, i.e. by GPs in primary care or in-hospital HF clinics, which supports prior findings from the COACH-2 study and NorthStar,^31^ which showed no differences in mortality and morbidity in patients followed-up by GP and HF clinic. Our study suggests even if they are followed up by a GP, patients can keep performing good self-care; meanwhile patients’ poor self-care behaviour may not be improved despite being followed-up at an HF clinic because those patients need additional interventions as described above. Decreasing self-care behaviour was seen in patients followed-up by both GPs and HF clinics, and it was a predictor for rehospitalisation as well as a marker of psychological distress. We therefore recommend that healthcare professionals assess patients’ self-care behaviour at least once or twice a year, and refer the patient to a specialist team if needed, regardless of the follow-up mode. It has also been found that implementing nurse-led HF clinics in primary care ensures evidence-based care throughout the chain of care.^32^ This model of follow-up has been associated with reduced hospital care use, improved adherence by health care providers to prescribing and evidence-based HF treatment as well as high patient satisfaction with care.^32^

### Limitation of the current study

There are some limitations in the study. First, the study is secondary analysis of the COACH-2 study and included clinically stable patients with systolic dysfunction and excluded HF patients who died during the follow-up in the current study. Therefore, the generalisability of the study results is limited. Impacts of self-care trajectories on clinical outcomes and the related factors HF might have been different among elderly patients with HF and preserved EF and patient with more severe HF. Despite that we included a relatively high number of patients in the study; the results could have been impacted by patients who were lost to follow-up due to death and frailty. Also in other long-term studies in this patient population there has been several drop-outs and can probably be explained by the natural trajectory of the disease with a poor prognosis. Patients often become more affected by the disease each year and there is a potential bias that the patients who are suffering the most do not cope to participate in long-term studies and respond to follow-up questionnaires.

In this type of complex interventions, it is always a concern not to have chosen sensitive outcomes that mirror the content of the intervention. Therefore, we used a variation of outcomes as recommended for complex interventions. A HF-specific scale was used to assess self-care behaviour in the study. Given the fact that many patients were hospitalised due to other CV or non-CV reasons, other self-care behaviours might have an important role in the study.

Lastly, we acknowledge limitations of our analysis in which patients who did not respond to the questionnaire were assumed to have poor self-care behaviour (i.e. EHFScBS <70). Some of the non-responding patients might have good self-care behaviour. However, we would like to make the best use of the patient data. In our subgroup analysis excluding the non-responding patients, decreased self-care behaviour remained an independent predictor for CV hospitalisation, but no factors influencing on self-care trajectory were identified. This might be in part due to small sample size. Further study is necessary to understand mechanism of changes of self-care behaviour among HF patients.

## Conclusion

There are a considerable number of patients (21%) who consistently have a poor self-care behaviour, even after follow-up by a HF clinic or primary care. In total, 27% decreased their level of self-care at 12 months and this decrease in self-care was related to worse outcomes, such as hospitalisations, increased depressive symptoms and reduced perceived control. Healthcare professionals need to repeatedly assess changes in self-care, as poor self-care behaviour might not be improved over time through the standard approach by HF nurses and primary care.

## Supplemental Material

10.1177_1474515120902317_Supplementary_Table – Supplemental material for Trajectory of self-care behaviour in patients with heart failure: the impact on clinical outcomes and influencing factors Click here for additional data file. Supplemental material, 10.1177_1474515120902317_Supplementary_Table for Trajectory of self-care behaviour in patients with heart failure: the impact on clinical outcomes and influencing factors by Maria Liljeroos, Naoko P Kato, Martje HL van der Wal, Maaike Brons, Marie Louise Luttik, Dirk J van Veldhuisen, Anna Strömberg and Tiny Jaarsma in European Journal of Cardiovascular Nursing
