# Supplementary material for: Trajectory of self-care behaviour in patients with heart failure: the impact on clinical outcomes and influencing factors
Source: Eur J Cardiovasc Nurs. 2020 Jan 29;19(5):421–32. doi: 10.1177/1474515120902317 (PMC7272123; doi:10.1177/1474515120902317)
Supplement: 10.1177_1474515120902317_Supplementary_Table – Supplemental material for Trajectory of self-care behaviour in patients with heart failure: the impact on clinical outcomes and influencing factors [file 10.1177_1474515120902317_Supplementary_Table.pdf]

Supplementary table. Characteristics of patients classified by changes of self-care behaviour (N=167)

	Poor-Poor (n=34)	Good-Poor (n=46)	Poor-Good (n=17)	Good-Good (n=70)	P-value
Demographics					
Education, n (%)					0.332
Basic education, 6 years	11 (34%)	9 (20%)	4 (24%)	17 (25%)	
Education after basic school	18 (56%)	30 (68%)	13 (76%)	39 (56%)	
University or higher professional education	3 (9.3%)	5 (11%)	0 (0%)	13 (19%)	
Clinical characteristics					
Admission in past 6 months, n (%)	3 (8.3%)	5 (11%)	1 (5.9%)	5 (7.1%)	0.933
Heart rate (bpm)	73.0±18.1	70.5±9.4	67.5±11.9	68.5±14.9	0.397
NT-pro BNP (ng/L)					
At baseline	753 (359-2124)	771 (330-2198)	1025 (203-1691)	1031 (440-1773)	0.927
Follow up	551 (279-1566)	829 (324-2198)	1155 (165-1707)	930 (296-1796)	0.810
Atrial fibrillation, n (%)	13 (36%)	18 (39%)	5 (29%)	32 (46%)	0.577
COPD, n (%)	6 (17%)	9 (20%)	1 (5.9%)	12 (17%)	0.664

Abbreviations: SD, standard deviation; COPD, chronic obstructive pulmonary disease.
